# Supplementary material for: Identification of photoperiod-induced specific miRNAs in the adrenal glands of Sunite sheep (Ovis aries)
Source: Front Vet Sci. 2022 Jul 22;9:888207. doi: 10.3389/fvets.2022.888207 (PMC9354845; doi:10.3389/fvets.2022.888207)
Supplement: Supplementary file 1 [file Data_Sheet_1.ZIP › Supplementary file/miRDeep2 file.zip!/miRDeep2 file/Know_Structure/SP42A3/oar-mir-382.pdf]

miRBase precursor : oar-mir-382
Total read count : 2617
oar-miR-382-5p read count : 980
oar-miR-382-3p read count : 1635
remaining reads : 2

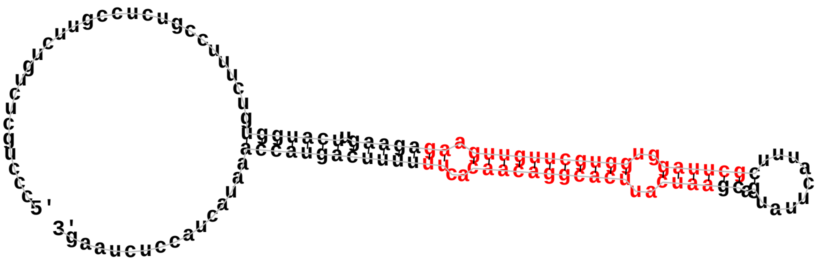

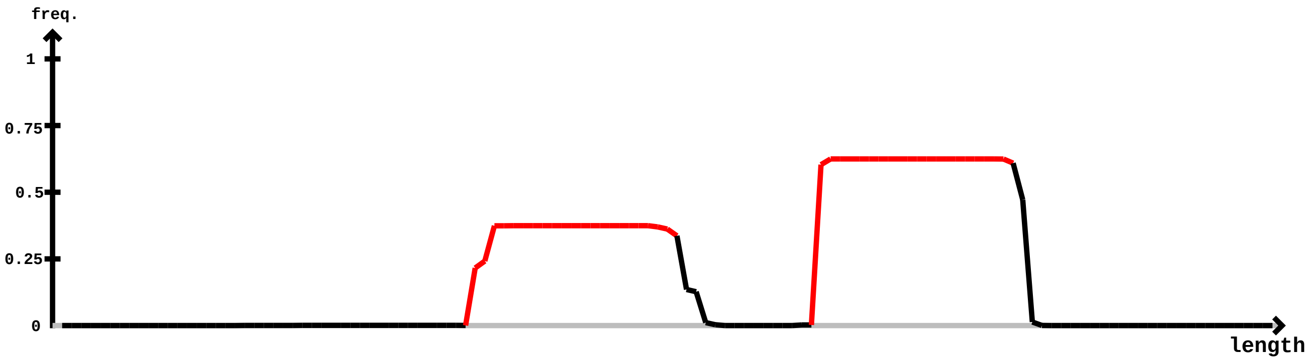


```
                                                                                              oar-miR-382-3p
                                         oar-miR-382-5p
5'- cccugcucugucuugccucugccuuucugugguacuugaagagaaguuguucgugguggauucgcuuuacuuaugacgaaucauucacggacaacacuuuuuucaguaccaaauacuaccucuaag  -3'   exp
    ....((........))...............(((((((.(((((((.(((((((((((..(((((((.........).))))))..)))))))))))..))))))))))))))...............   reads  mm
    ..................cugccuuucugugguacuugaag.......................................................................................   1      0
    ........................uucugugguacuugaaga......................................................................................   1      0
    ............................................gaaguuguucgugguggau.................................................................   12     0
    ............................................gaaguuguucgugguggauu................................................................   20     0
    ............................................gaaguuguucgugguggauuc...............................................................   63     0
    ............................................gaaguuguucgugguggauucg..............................................................   457    0
    ............................................gaaguuguucgugguggauucgc.............................................................   12     0
    ............................................gaaguuguucgugguggauucgcuuu..........................................................   1      0
    .............................................aaguuguucgugguggauu................................................................   1      0
    .............................................aaguuguucgugguggauuc...............................................................   2      0
    .............................................aaguuguucgugguggauucg..............................................................   62     0
    .............................................aaguuguucgugguggauucgc.............................................................   3      0
    ..............................................aguuguucgugguggauuc...............................................................   1      0
    ..............................................aguuguucgugguggauucg..............................................................   8      0
    ..............................................aguuguucgugguggauucgc.............................................................   7      0
    ..............................................aguuguucgugguggauucgcu............................................................   303    0
    ..............................................aguuguucgugguggauucgcuu...........................................................   20     0
    ..............................................aguuguucgugguggauucgcuuu..........................................................   7      0
    ................................................uuguucgugguggauucgcuu...........................................................   1      0
    ..............................................................................cgaaucauucacggacaacacu............................   2      0
    ..............................................................................cgaaucauucacggacaacacuu...........................   4      0
    ................................................................................aaucauucacggacaacac.............................   1      0
    ................................................................................aaucauucacggacaacacu............................   37     0
    ................................................................................aaucauucacggacaacacuu...........................   351    0
    ................................................................................aaucauucacggacaacacuuu..........................   1161   0
    ................................................................................aaucauucacggacaacacuuuu.........................   22     0
    ................................................................................aaucauucacggacaacacuuuuu........................   1      0
    .................................................................................aucauucacggacaacacu............................   1      0
    .................................................................................aucauucacggacaacacuu...........................   4      0
    .................................................................................aucauucacggacaacacuuu..........................   39     0
    .................................................................................aucauucacggacaacacuuuu.........................   12     0
```
